# The Effect of Conservative Oxygen Therapy in Reducing Mortality in Critical Care Patients: A Meta-Analysis and Trial Sequential Analysis

**DOI:** 10.3389/fmed.2021.738418

**Published:** 2021-12-10

**Authors:** Yue-Nan Ni, Ting Wang, Bin-Miao Liang, Zong-An Liang

**Affiliations:** Department of Respiratory and Critical Care, West China School of Medicine, West China Hospital, Sichuan University, Chengdu, China

**Keywords:** conservative oxygen therapy, mortality, meta-analysis, critically ill, trial sequence analysis

## Abstract

**Background:** Conservative oxygen therapy can prevent both hypoxemia and hyperoxemia, but the effect on the prognosis of patients admitted to the intensive care unit (ICU) remains controversial.

**Methods:** All controlled studies comparing conservative oxygen therapy and conventional oxygen therapy in adult patients admitted to the ICU were searched. The primary outcome was mortality, and the secondary outcomes were length of ICU stay (ICU LOS), length of hospital stay (hospital LOS), length of mechanical ventilation (MV) hours, new organ failure during ICU stay, and new infections during ICU stay.

**Results:** Nine trials with a total of 5,759 patients were pooled in our final studies. Compared with conventional oxygen therapy, conservative oxygen therapy did not reduce overall mortality (Z = 0.31, *p* = 0.75) or ICU LOS (Z = 0.17, *p* = 0.86), with firm evidence from trial sequential analysis, or hospital LOS (Z = 1.98, *p* = 0.05) or new infections during the ICU stay (Z = 1.45, *p* = 0.15). However, conservative oxygen therapy was associated with a shorter MV time (Z = 5.05, *p* < 0.00001), reduction of new organ failure during the ICU stay (Z = 2.15, *p* = 0.03) and lower risk of renal replacement therapy (RRT) (Z = 2.18, *p* = 0.03).

**Conclusion:** Conservative oxygen therapy did not reduce mortality but did decrease MV time, new organ failure and risk of RRT in critically ill patients.

**Systematic Review Registration:** identifier [CRD42020171055].

## Background

Hypoxemia is life threatening (1, 2) and is related to increasing intensive care unit (ICU) mortality (3). Oxygen administration is a life-saving treatment commonly used in patients admitted to the ICU (4, 5). Unfortunately, although oxygen administration in ICUs is recommended by many guidelines, the most suitable oxygenation target remains unknown (6).

Studies have shown that excess oxygen delivery is very common, and approximately 50% of patients show hyperoxemia, among whom 4% have severe hyperoxemia (7–9). In our previous study, hyperoxia was independently associated with ICU mortality in mechanical ventilation patients [odds ratio (OR) 1.22, 95% confidence interval (CI) 1.12–1.33] (10).

Thus, to prevent hypoxemia and avoid adverse events caused by hyperoxemia, some researchers have studied conservative oxygen therapy, which adheres to the pulse oxygen saturation (SpO_2_) goal between 88 and 92% with the lowest fraction of inspired oxygen (FiO_2_). However, the results have remained controversial. In the study by Girardis et al., which included 434 patients, conservative oxygen therapy reduced ICU mortality by approximately 19% (*p* = 0.01) (11). However, in the study by Mackle et al., conservative oxygenation targets did not show any advantages in ICU mortality over the conventional oxygenation target (35.7 vs. 34.5%) (12).

Therefore, based on the controversial results of the effects of conservative oxygen therapy, we conducted a systematic review with meta-analysis and trial sequence analysis of all published trials aiming to identify the role of conservative oxygen therapy in improving the outcomes of patients admitted to the ICU.

## Methods

### Search Strategies

We systematically searched PubMed, Embase, Medline, Cochrane Central Register of Controlled Trails (CENTRAL), and Information Sciences Institute (ISI) Web of Science using the keywords “conservative oxygen therapy” or “conservative oxygenation target” or “oxygenation target” and “critically ill” or “ICU” or “intensive care unit” without limitations on the publication type or language from 1946 to August 2020. A hand search through the reference lists of relevant primary and review articles was also performed for completeness.

### Inclusion and Exclusion Criteria

Eligible clinical trials were identified based on the following criteria: (1) the subjects enrolled in each study included patients admitted to the ICU; (2) patients were divided into an experimental group, in which conservative oxygen therapy (the target of fraction of inspired oxygen (FiO_2_) including in room air, or arterial partial pressure of oxygen (PaO_2_), or arterial oxygen saturation (measured by blood analysis), or peripheral oxygen saturation [measured by a pulse oximeter (SpO_2_)] were lower than the control group) was applied, and a control group (higher oxygen target); and (3) outcomes included, but were not limited to, mortality at the longest following up time point, length of ICU stay (ICU LOS) (since the start of the study), length of hospital stay (hospital LOS) (since the start of the study), length of mechanical ventilation (MV) hours (since the start of the study), new infection (defined as positive bacterial culture in sputum, urine, or blood),new organ failure (defined as a SOFA score ≥3 for the corresponding organ occurring 48 h or more after ICU admission) during ICU and the rate of renal replacement therapy (RRT). We excluded studies if they were performed on animals or in patients younger than 18 years old or were published as reviews or case reports.

### Study Selection

Two investigators (Y-NN and TW) independently screened studies for eligibility according to predefined study selection criteria. Titles and abstracts from the search were examined, and full texts were obtained for all potentially relevant records. Any disagreement was resolved through discussion with a third author (B-ML).

### Data Extraction

Data were extracted in duplicate by two independent data collectors using a standard form recommended by Cochrane. Authors, publication year, study design, country, NCT No., population, demographic characteristics (age, gender, etc.), disease conditions [the Acute Physiologic and Chronic Health Evaluation III (APACHE III) and Simplified Acute Physiologic Score II (SAPS II)], outcome measures, and study results were extracted. In cases in which data points were missing or ambiguously reported, the corresponding author was contacted by email to obtain the data.

### Quality Assessment

Two authors independently assessed the risk of systematic bias of trials included in the meta-analysis according to the Cochrane Handbook (13). Disagreement during the review process was resolved by consensus through involvement of a third review author. Risk of bias was rated according to the following domains: (1) sequence generation; (2) allocation concealment; (3) blinding of participants and personnel; (4) blinding of outcome assessment; (5) incomplete outcome data; (6) selective outcome reporting; and (7) other sources of bias (specifically including baseline imbalance, early stopping and financial bias).

### Statistical Analysis

Meta-analysis and forest plots were prepared using the Cochrane systematic review software Review Manager (RevMan; version 5.3.5; The Nordic Cochrane Center, The Cochrane Collaboration, Copenhagen, Denmark, 2014). We used the Mantel-Haenszel model to verify the hypothesis and rendered statistical significance as a Z-value and *p* < 0.05. I^2^ was used to estimate variation across studies attributable to heterogeneity. A value of *p* < 0.1 and I^2^ >50% indicated significance. A random-effects model was applied in the presence of statistical heterogeneity; for continuous data, we calculated the mean difference (MD) and 95% CIs, while for dichotomous data, we calculated the ORs and 95% CIs. We also performed sensitivity analysis to substitute alternative decisions or ranges of values for decisions that were arbitrary or unclear. Trial sequential analysis was performed using TSA viewer software, version 0.9, to correct for cumulative heterogeneous results, decrease type I error and model the potential effects of uncompleted registered studies. Information size was computed assuming an alpha risk of 5% and a beta risk of 20%. Trial sequential analysis 95% CI boundaries that excluded the null (<1.00 or >1.00) were considered statistically significant.

## Results

Initially, 1,570 records were identified, of which 1,567 were extracted from electronic databases, and three were extracted from reference list reviews. By screening the titles and abstracts, 1,561 studies were discarded due to duplication (*n* = 1,297), animal experiments (*n* = 210) and non-adult patients (*n* = 52). We researched the full-text articles for the remaining 11 studies, and nine trials were eventually enrolled in our final analysis because one study did not report related outcomes, and one was not designed as expected (Supplementary Figure 1).

### Study Description

All nine studies compared the outcomes of conservative oxygen therapy alone with those of conventional oxygen therapy (11, 12, 14–20). Six studies were randomized, controlled trials (RCTs) (11, 12, 16, 18–20), one study was a retrospective nest cohort analysis (14), and the other two studies were prospective before-after studies (15, 17). Mortality was reported in nine studies (11, 12, 14–20), among which hospital mortality was reported in three studies (11, 14, 15), ICU mortality was reported in four studies (11, 14, 15, 19), 28-day mortality was reported in four studies (14, 17–19), 30-day mortality was reported in one study (15), 90-day mortality was reported in five studies (12, 16, 18–20), and 180-day mortality was reported in one study (15). ICU LOS was presented in six studies (11, 12, 14–16, 18), Hospital LOS was reported in five studies (11, 12, 14–16). MV hours was reported in four studies (12, 14, 15, 19). The rate of new organ failure was recorded in two studies (11, 17), the rate of new infection was recorded in three studies (11, 17, 19), and the rate of renal replacement therapy was reported in five studies (12, 15, 17, 18, 20). Details of each study are summarized in Table 1.

**Table 1:** Characteristics of included studies.

**Study ID**	**Study design**	**NCT No**.	**Country**	**Population**	**Diagnosis**	**Conservative group**	**Conventional group**	**Target conservative**	**Target conventional**	**Length of exposure**
Asfar et al. (18)	multicenter, randomized trial	NCT01722422	France	434	Septic shock	SpO_2_ 88–95%	FiO_2_ 100% for first 24 hr	Patients with hyperoxemia (>120 mmHg):121 (56%)	Patients with hyperoxemia (>120 mmHg):110 (51%)	24 h
Barrot et al. (19)	multicenter, randomized trial	NCT02713451	France	201	Acute respiratory distress syndrome	SpO_2_ 88–92% PaO_2_ 55–70 mmHg	SpO_2_ ≥96% PaO_2_ 90–105 mmHg	4.18% <55.0 mm Hg 21.00% >70.0 mm Hg	27.45% <90.0 mm Hg 24.02% >105.0 mm Hg	7 days
Eastwood et al. (14)	Retrospective nested cohort study	NCT 01684124	Australia	100	Cardiac arrest	SpO_2_ 88–92%	Oxygenation target was prescribed by their doctors	Hyperoxemia time (>120 mmHg): 28%	Hyperoxemia time (>120 mmHg): 66%	During MV
Esatwood et al. (15)	uncontrolled before-and-after study	ACTRN1261300 1322729	Australia	543	Cardiac surgery	SpO_2_ 88–92%	Oxygenation target was prescribed by their doctors	Mean PaO_2:_88 mmHg (81–96)	Mean PaO_2:_104 mmHg (89–121)	During MV
Girardis et al. (11)	Randomized controlled trial	NCT01319643	Italy	434	Medical, surgical	SpO_2_ 94–98%	SpO_2_ 97–100%	Mean PaO_2:_87 mmHg (79–97)	Mean PaO_2:_102 mmHg (88–116)	During ICU stay
Mackle et al. (12)	Randomized controlled trial	ACTRN126150 00957594	Australia and New Zealand	965	Mixed	Least FiO_2_ to guarantee 97%>SpO_2_>90%, minimize exposure to SpO_2_ <97%	FiO_2_ >0.3, no upper limit	Median number of h per patient SpO_2_ ≥97%: 27 [11–63.5]	Median number of h per patient SpO_2_ ≥97%: 49 [22–112]	During ICU stay
Panwar et al. (16)	Multicenter randomized controlled trial	ACTRN126130 00505707	Australia, New Zealand, and France	103	Trauma, medical, surgical	SpO_2_ 88–92%	SpO_2_ ≥96%	Mean PaO_2:_70 mmHg (68–73)	Mean PaO_2:_92 mmHg (89–96)	During MV
Schjørring et al. (20)	Multicenter randomized controlled trial	NCT03174002	Denmark, Switzerland, Finland, the Netherlands, Norway, the United Kingdom, and Iceland	2,888	Pneumonia, multiple trauma, hemorrhagic or ischemic stroke, traumatic brain injury, myocardial infarction, intestinal ischemia, cardiac arrest, ARDS	PaO_2_ 60 mmHg	PaO_2_ 90 mmHg	Median PaO_2_:70.8 (66.6–76.5) mmHg	Median PaO_2:_93.3 (87.1–98.7) mmHg	Up to 90 days
Suzuki et al. (17)	Pilot prospective before-and-after study	NCT 01684124	Australia	105	Cardiovascular, gastrointestinal, neurological impairment, surgical procedure, others	SpO_2_ 90–92%	Oxygenation target was prescribed by their doctors	Mean time weighted average PaO_2:_83 mmHg (71–94)	Mean time weighted average PaO_2:_107 mmHg (94–131)	During MV

*ARDS, acute respiratory distress syndrome; FiO_2_, fraction of inspired oxygen; NR, not reported; PaO_2_, partial pressure of oxygen in the arterial; SpO_2_, pulse oxygen saturation*.

A total of 5,759 patients were pooled from all of the included trials in our final systematic review and meta-analysis, among whom 2,903 patients were treated with conservative oxygen therapy, and 2,856 patients received conventional oxygen therapy. Details of the baseline characteristics of the patients in each enrolled study are shown in Table 2.

**Table 2:** Baseline characteristics of patients.

	**Conservative oxygen therapy**					**Conventional oxygen therapy**				
**Study ID**	**Age, years mean (SD)**	**Male** ***n***, **(%)**	**SAPS II mean (SD)**	**APACHE III mean (SD)**	**SOFA median (IQR)**	**Age, years mean (SD)**	**Male** ***n***, **(%)**	**SAPS II mean (SD)**	**APACHE III mean (SD)**	**SOFA median (IQR)**
Asfar et al. (18)	66.3 (14.6)	140 (65%)	72.5(11.1) (SAPSIII)^a^	NR	10.3 ± 2.9	67.8 (12.7)	137 (63%)	71.6 (11.1) (SAPSIII)^a^	NR	10.2 ± 2.7
Barrot et al. (19)	63 (15.5)^a^	65 (65.7%)^c^	66.9 (13.7) (SAPSIII)^a^	NR	9.3 ± 3.68	63.5 (14.5)^a^	64 (62.7%)^c^	67.9 (14.4) (SAPSIII)^a^	NR	8.9 ± 3.6
Eastwood et al. (14)	67 (59–77)^b^	29 (58%)^c^	NR	121 (105–142)^b^	NR	65 (50–71)^b^	34 (68%)^c^	NR	125 (107–141) ^b^	NR
Eastwood et al. (15)	65 (56–73)^b^	209 (70.1%)^c^	NR	NR	NR	67 (59–74)^b^	179 (73.1%)^c^	NR	NR	NR
Girardis et al. (11)	63 (51–74)^b^	121 (56%)^c^	37 (26–49)^b^	NR	NR	65 (52–76)^b^	125 (57.3%)^c^	39 (28–55)^b^	NR	NR
Mackle et al. (12)	58.1 (16.2)^a^	306 (63.2%)^c^	NR	23.6 (9.3) (APACHEII)^a^	NR	57.5 (16.1)^a^	302 (62.8%)^c^	NR	23.3 (9.4) (APACHEII)^a^	NR
Panwar et al. (16)	62.4 (14.9)^a^	32 (62%)^c^	NR	79.5 (61–92.5)^b^	7.9 ± 2.9	62.4 (17.4)^a^	33 (65%)^c^	NR	70 (50–84)^b^	7.4 ± 3.1
Schjørring et al. (20)	70 (60–77)	925 (63.7%)	NR	NR	9 (8–11)	70 (60–77)	946 (64.9%)	NR	NR	9 (8–11)
Suzuki et al. (17)	56 (16)^a^	32 (59%)^c^	NR	62 (49–92)^b^	NR	59 (17)^a^	38 (75%)^c^	NR	68 (42–94)^b^	NR

*APACHE, The Acute Physiologic and Chronic Health Evaluation; IQR, interquartile range; NR, not report; SAPS Simplified Acute Physiologic Score; SD, standard derivation*.

a *mean (SD)*.

b *median (IQR)*.

c *n (%)*.

### Quality Assessment

Quality assessment of the nine enrolled studies showed that there was no bias in attrition or reporting in nine studies but high bias existed in performance in nine studies and in selection and detection in three studies. No studies were excluded for low quality or dubious decisions in the sensitivity analysis (Supplementary Figures 2, 3).

### Heterogeneity

No significant statistical heterogeneity was found in overall mortality between the conservative and conventional groups (I^2^ = 46%, χ^2^ = 14.74, *p* = 0.06), in ICU LOS (I^2^ = 25%, χ^2^ = 6.63, *p* = 0.25), hospital LOS (I^2^ = 0%, χ^2^ = 3.39, *p* = 0.49), length of MV hours (I^2^ = 37%, χ^2^ = 4.78, *p* = 0.19), or new organ failure during the ICU stay (I^2^ = 0%, χ^2^ = 0.05, *p* = 0.82), or in new infections during the ICU stay (I^2^ = 2%, χ^2^ = 3.08, *p* = 0.38).

### Mortality

**Figure 1:**
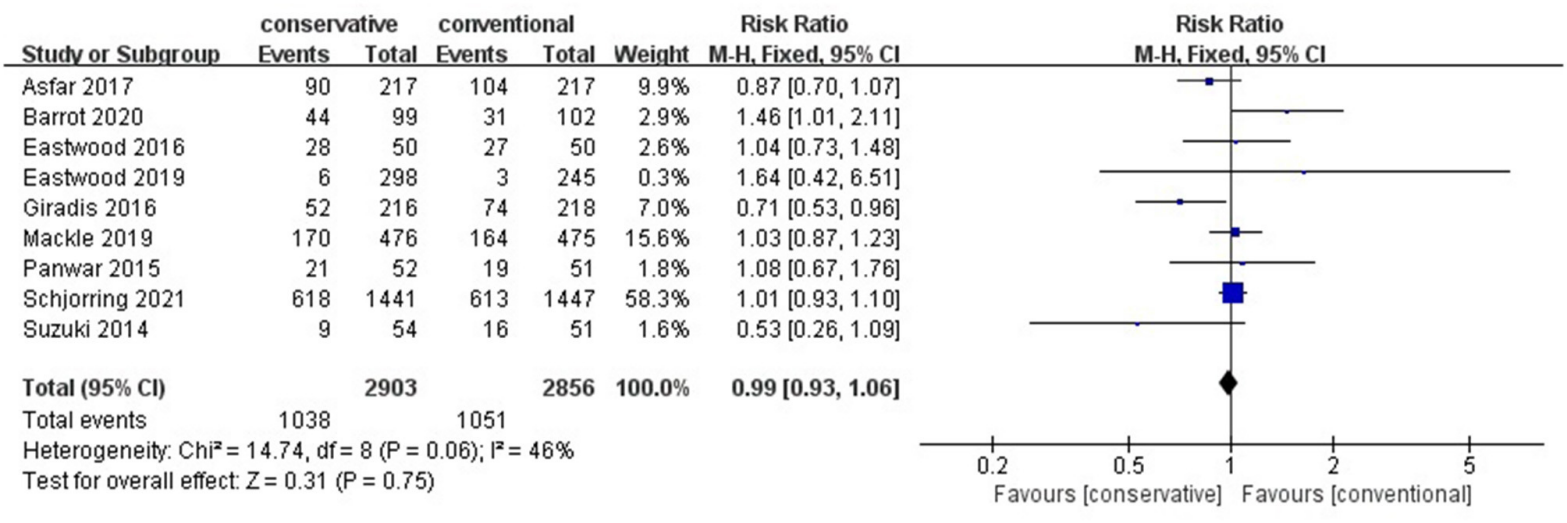
Overall mortality. CI, confidence interval; SD, standard deviation.

**Figure 2:**
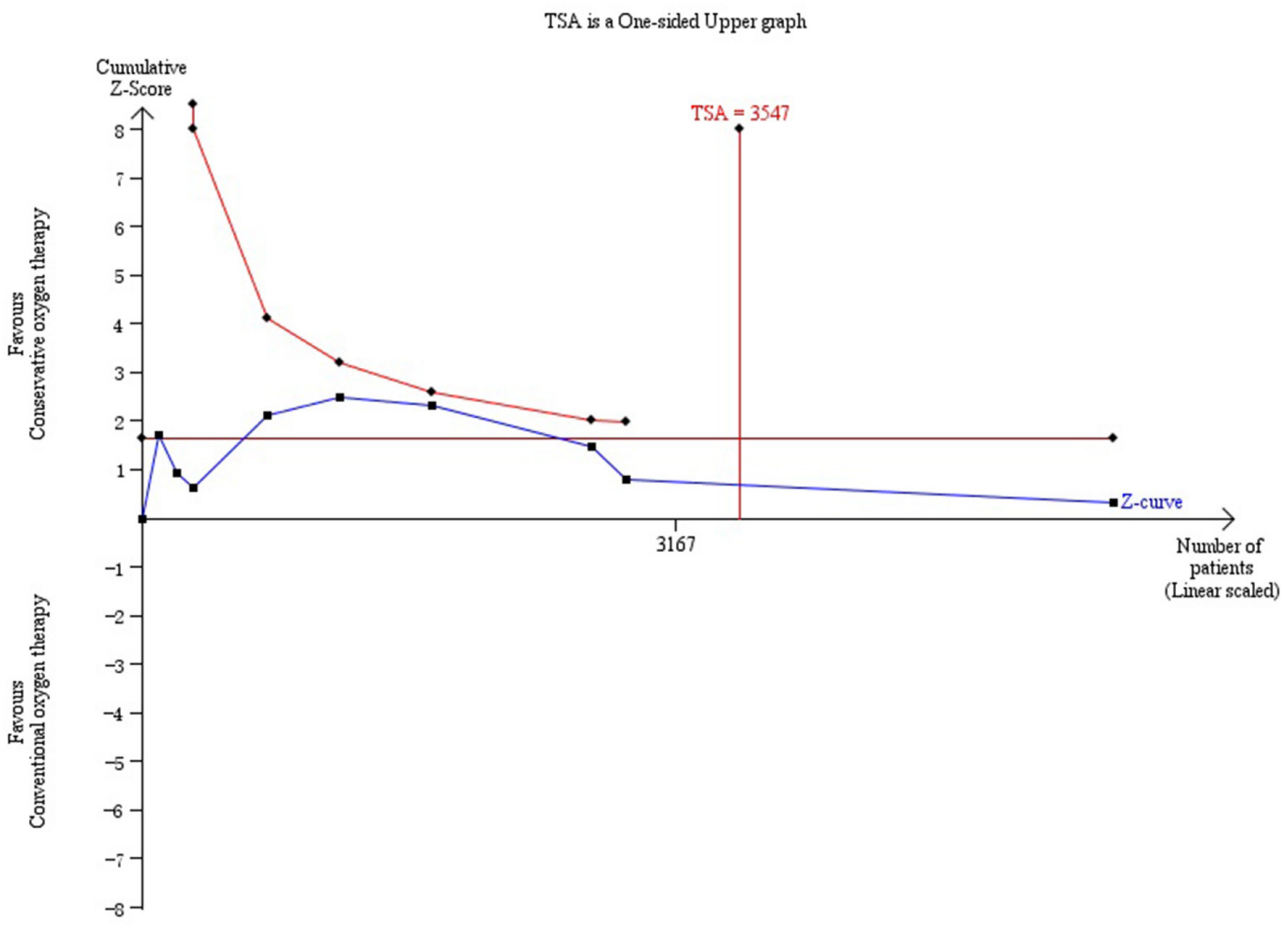
Trial sequential analysis of mortality. TSA, trial sequential analysis.

No significant difference in overall mortality was found with conservative oxygen therapy compared with conventional oxygen therapy (RR 0.99, 95% CI 0.93–1.06; Z = 0.31, *p* = 0.75) (Figure 1) or in ICU mortality (RR 0.98, 95% CI 0.64–1.49;Z = 0.10, *p* = 0.92), (Supplementary Figure 4) hospital mortality (RR 0.90, 95% CI 0.59–1.39; Z = 0.46, *p* = 0.65), (Supplementary Figure 5) 28-day mortality (RR 0.91, 95% CI 0.76–1.08; Z = 1.07, *p* = 0.28) (Supplementary Figure 6) or 90-day mortality (RR 1.03, 95% CI 0.92–1.15;Z = 0.48, *p* = 0.63) (Supplementary Figure 7). We performed subgroup analyses of all of the randomized controlled studies, and no advantages of conservative oxygen therapy were found (RR 0.98, 95% CI 0.86–1.12;Z = 0.26, *p* = 0.80) (Supplementary Figure 8). Moreover, we performed subgroup analysis (Supplementary Figure 9) and included four studies that defined conservative oxygen therapy as SpO_2_ 88–92%, and the same result was found (RR 1.03, 95% CI 0.95–1.11; Z = 0.65, *p* = 0.52). The results were confirmed by the TSA test, and the required information size was reached (Figure 2).

### ICU LOS

**Figure 3:**
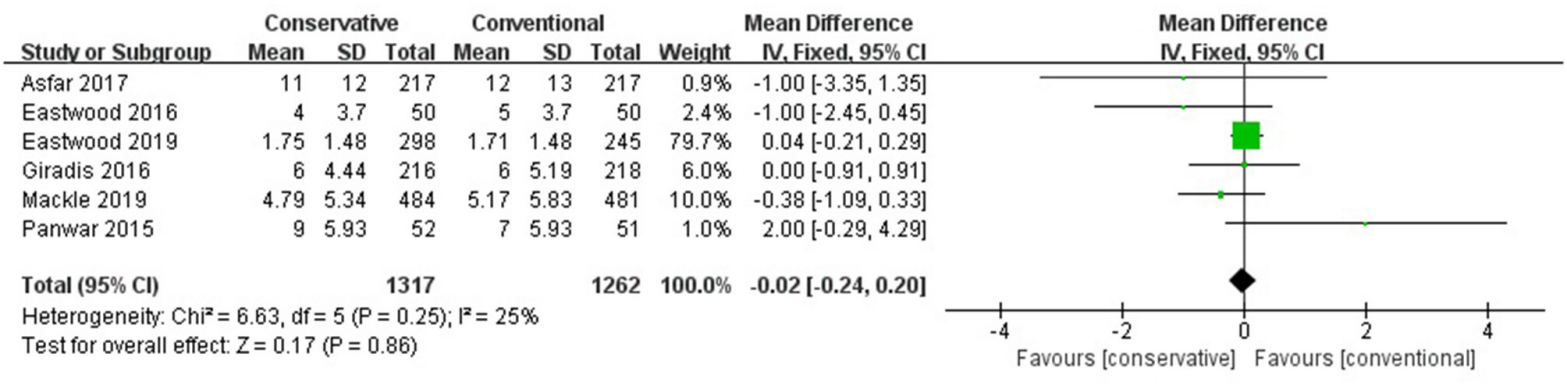
ICU LOS. CI, confidence interval; ICU, intensive care unit; LOS, length of stay; MD, mean difference; SD, standard deviation.

Figure 3 shows that the difference was not significant between conservative oxygen therapy and conventional oxygen therapy (MD −0.02, 95% CI −0.24–0.20; Z = 0.17, *p* = 0.86) in ICU LOS.

### Hospital LOS

**Figure 4:**
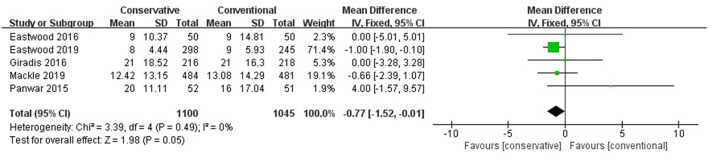
Hospital LOS. CI, confidence interval; LOS, length of stay; MD, mean difference; SD, standard deviation.

No significant role of conservative oxygen therapy in hospital LOS was found (MD −0.77, 95% CI −1.52– −0.01, Z = 1.98, *p* = 0.05) (Figure 4).

### MV Hours

**Figure 5:**
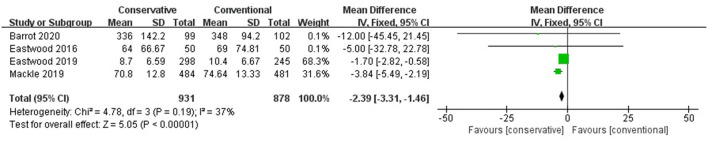
MV hours. CI, confidence interval; MD, mean difference; MV, mechanical ventilation; SD, standard deviation.

Conservative oxygen therapy reduced the MV hours compared with conventional oxygen therapy (MD −2.39, 95% CI −3.31– −1.46; Z = 5.05, *p* < 0.001) and prolonged the MV-free days (MD 683 683 0.96, 95% CI 0.55–1.37; Z = 4.61, *p* < 0.001) (Figure 5).

### New Organ Failure During ICU Stay

**Figure 6:**
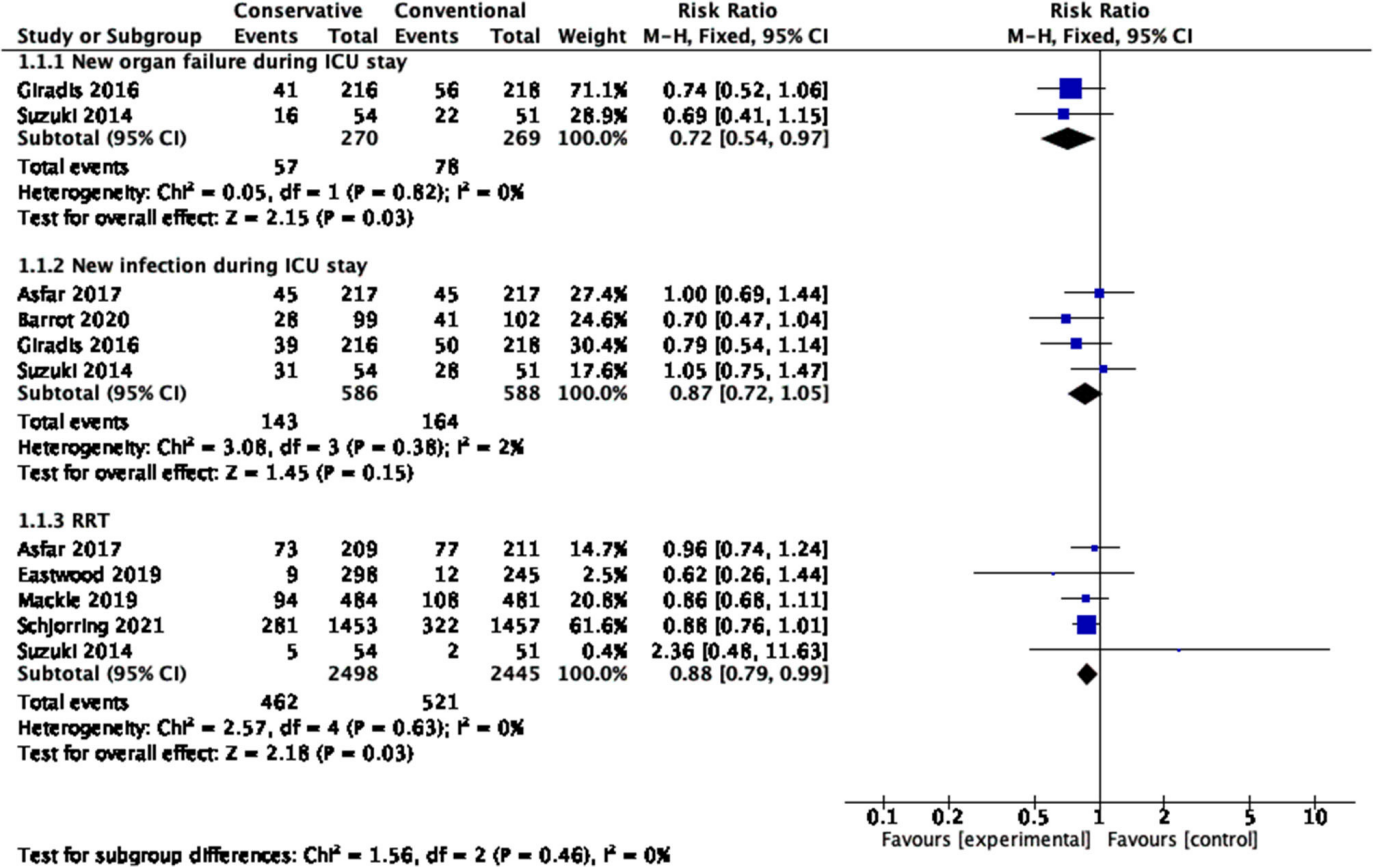
New infection, new organ failure and rate of RRT during the ICU stay. CI, confidence interval; ICU, intensive care unit; RRT, renal replacement therapy; SD, standard deviation.

Figure 6 shows that differences in new organ failure during the ICU stay existed in the comparison between conservative oxygen therapy and conventional oxygen therapy (RR 0.72, 95% CI 0.54–0.97; Z = 2.15, *p* = 0.03).

### New Infection During ICU Stay

No significant differences in new infections during the ICU stay existed between conservative oxygen therapy and conventional oxygen therapy (RR 0.87, 95% CI 0.72–1.05; Z = 1.45, *p* = 0.15) (Figure 6).

### RRT

Figure 6 shows that conservative oxygen therapy reduced the risk of renal replacement therapy (RR 0.88, 95% CI 0.79−0.99; Z = 2.18, *p* = 0.03).

## Discussion

In our meta-analysis, we found that conservative oxygen therapy did not decrease the rate of mortality, ICU LOS, hospital LOS or new infections during the ICU stay in critically ill patients. However, conservative oxygen therapy could decrease the MV time, new organ failure during the ICU stay and the risk of RRT.

The advantages of conservative oxygen therapy should be addressed. Conventional therapy would leave 44.5% of patients exposed to hyperoxemia, compared to only approximately 11.4% in the conservative oxygen group (15). The disadvantages of hyperoxemia have been well demonstrated by many studies. First, high inspired oxygen concentrations inhibit the immune system, compromising the ability of macrophages (21), decreasing the abundance of immunoregulatory populations, (22) causing structural changes within alveolar macrophages and leading to serious impairment of their antimicrobial activity (23, 24). Second, pulmonary injury is induced by hyperoxemia. As mentioned above, hyperoxemia can result in decreased mucociliary clearance, atelectasis, inflammation, pulmonary edema, and eventually interstitial fibrosis (25, 26). The combination of immune system compromise and pulmonary injury is related to a higher risk of ventilator-associated pneumonia (VAP). A retrospective, observational study of 503 enrolled patients showed that both hyperoxemia at ICU admission and the percentage of days with hyperoxemia were independently associated with VAP (27). As studies have shown, the rate of VAP is associated with a longer MV period (28). Moreover, two of the enrolled studies showed a trend toward lower use of mandatory MV mode in the conservative oxygen group, which might indicate earlier attempts to wean patients in response to lower FiO_2_ requirements. This is one of the reasons for the significantly shorter MV hours in the conservative oxygen therapy group (14, 16). Third, every organ, not only the lung, would be damaged by the production of reactive oxygen species (ROS) resulting from high concentrations of oxygen. ROS-mediated stress can lead to cellular necrosis and apoptosis (29). In addition, oxidative stress is responsible for direct damage to biological molecules and indirect injury through the release of cytotoxic products and the mutagenic effects of lipid oxidation (30). ROS-mediated stress and oxidative stress caused by high inspired oxygen concentrations could promote systemic organ failure; otherwise, the decrease of ROS in the conservative oxygen therapy group would lead to less new organ failure during the ICU stay. Hyperoxia exacerbates renal dysfunction, which is mediated by oxygen radical-related injury (31), which is also the one of the reasons that conservative oxygen therapy reduces the rate of RRT.

However, despite the advantages of conservative oxygen therapy, lower mortality, shorter ICU LOS and shorter hospital LOS were not found in our study. We believe that the following reasons might explain this outcome. First, many factors contribute to the mortality of patients. Although conservative oxygen therapy could bring some benefit to patients, many other factors, such as the severity of baseline disease, also contribute significantly to mortality, ICU LOS and hospital LOS (32). Thus, the benefit of conservative oxygen therapy could not be found when combined with so many factors. Meanwhile, in the analysis about mortality in the conservative oxygen therapy and conventional oxygen therapy, the cumulative z score had crossed the trial sequential monitoring boundary, suggesting further trials were not required. Second, conservative oxygen therapy actually exposes patients to higher risk of hypoxia while avoiding hyperoxemia. Hypoxia was also related to higher mortality (33). One of the included studies found that patients in the conservative oxygen group had a significantly higher risk of mesenteric ischemia events and a higher heart rate (19). Moreover, patients were at higher risk of cardiac adverse outcomes, although statistical significance was not attained. This result indicated that there were adverse outcomes of conservative oxygen therapy that could not be ignored and might offset its advantages. More than one study found that the influence of oxygenation on mortality was similar to a “U” shape (34, 35). The lowest mortality was found when the SpO_2_ was approximately 94 to 98%. Only in the study by Girardis et al. was the SpO_2_ of conservative oxygen therapy located at the lowest point of the “U” shape. Of all of the included studies, only the study by Girardis et al. also explained why it found the most significant effect of conservative oxygen therapy in reducing ICU mortality in all of the included studies (11). The definition of conservative oxygen therapy in published conservative oxygen therapy studies was not consistent. We recommend that further study carefully consider the target of oxygenation in conservative oxygen therapy. Third, the definitions of conservative oxygen therapy and conventional oxygen therapy overlapped to some extent between the included studies, rendering the advantages of conservative oxygen therapy unclear. Fourth, we found that the effect of conservative oxygen therapy was different in patients with different primary diseases. For example, the advantages of conservative oxygen therapy were obvious in patients with hypoxic ischemia, such as patients with cardiac arrest and hypoxic ischemic encephalopathy (14, 36) but it could add to the mortality of patients with ARDS (37). This result was also consistent with the conclusions of our previous study that hyperoxia will increase mortality, especially in patients with hypoxic ischemia (10). Therefore, we also recommend that further studies explore conservative oxygen therapy in different primary diseases.

In addition, we did not find any advantages of conservative oxygen therapy for new infections during the ICU stay compared with conventional oxygen therapy. The incidence of new infections might have been underestimated because only those ascertained by microbiological samples were recorded (11). Moreover, only two of the enrolled studies reported data about new infections during the ICU stay. Thus, the small sample might also be a limitation.

In one of the included studies, the role of conservative oxygen therapy in increasing MV-free days was found in suspected hypoxic ischemic encephalopathy but not in other diagnoses (12). In our previous study, we found that hyperoxia led to higher mortality in cardiac arrest patients (10). This outcome indicated that patients who have experienced hypoxemia might be more likely to benefit from conservative oxygen therapy. Moreover, in a previous meta-analysis of conservative oxygen therapy in acutely ill patients, more than half of the included studies enrolled patients who experienced hypoxemia due to stroke, myocardial infarction, etc. (37). We believe that this fact was the main reason why a previous analysis found advantages of conservative oxygen therapy in mortality, while our analysis did not.

There are also several limitations in our study that must be addressed. First, high clinical heterogeneity existed in our analysis. Also: (1) the primary diseases of patients included in our enrolled studies were mixed, and conservative oxygen therapy might have more benefit in hypoxic ischemic encephalopathy, but we could not perform the subgroup analysis due to lack of data; (2) the severity of patients who were admitted to the ICU also varied in the included studies; and (3) although all of the studies divided participants into conservative and conventional groups, the actual oxygenation level in each group varied in the included studies. In addition, because of the limit of FiO_2_ titration, there were episodes in which the oxygenation level of patients was out of the range of target oxygenation levels, which might influence the application of our conclusions.

## Conclusion

Compared with conventional oxygen therapy, conservative oxygen therapy had no effects on mortality, ICU LOS or hospital LOS in critically ill patients but could decrease the length of MV hours, new organ failure and risk of RRT.

## Data Availability Statement

The original contributions presented in the study are included in the article/Supplementary Material, further inquiries can be directed to the corresponding author/s.

## Author Contributions

Y-NN and TW designed the study, drafted the manuscript, and conducted the literature search and data analysis. B-ML and Z-AL made the decision to submit the report for publication. All authors read and approved the final manuscript.

## Funding

This study was partly supported by the National Key Research and Development Program of China (2016YFC1304303).

## Conflict of Interest

The authors declare that the research was conducted in the absence of any commercial or financial relationships that could be construed as a potential conflict of interest.

## Publisher's Note

All claims expressed in this article are solely those of the authors and do not necessarily represent those of their affiliated organizations, or those of the publisher, the editors and the reviewers. Any product that may be evaluated in this article, or claim that may be made by its manufacturer, is not guaranteed or endorsed by the publisher.

## Abbreviations

APACHE: The Acute Physiologic and Chronic Health Evaluation
CENTRAL: Cochrane Central Register of Controlled Trails
CI: confidence interval
FiO_2_: fraction of inspired oxygen
ICU: intensive care unit
ISI: Information Sciences Institute
LOS: length of stay
MD: mean difference
MV: mechanical ventilation
PaO_2_: partial pressure of arterial oxygen
RCT: randomized, controlled trial
OR: odds ratio
ROS: reactive oxygen species
SpO_2_: pulse oxygen saturation
SAPS: Simplified Acute Physiologic Score
SD: standard deviation.
