# Supplementary material for: The Effect of Conservative Oxygen Therapy in Reducing Mortality in Critical Care Patients: A Meta-Analysis and Trial Sequential Analysis
Source: Front Med (Lausanne). 2021 Dec 10;8:738418. doi: 10.3389/fmed.2021.738418 (PMC8702806; doi:10.3389/fmed.2021.738418)

Supplemental material

Figure 1s Study flow.

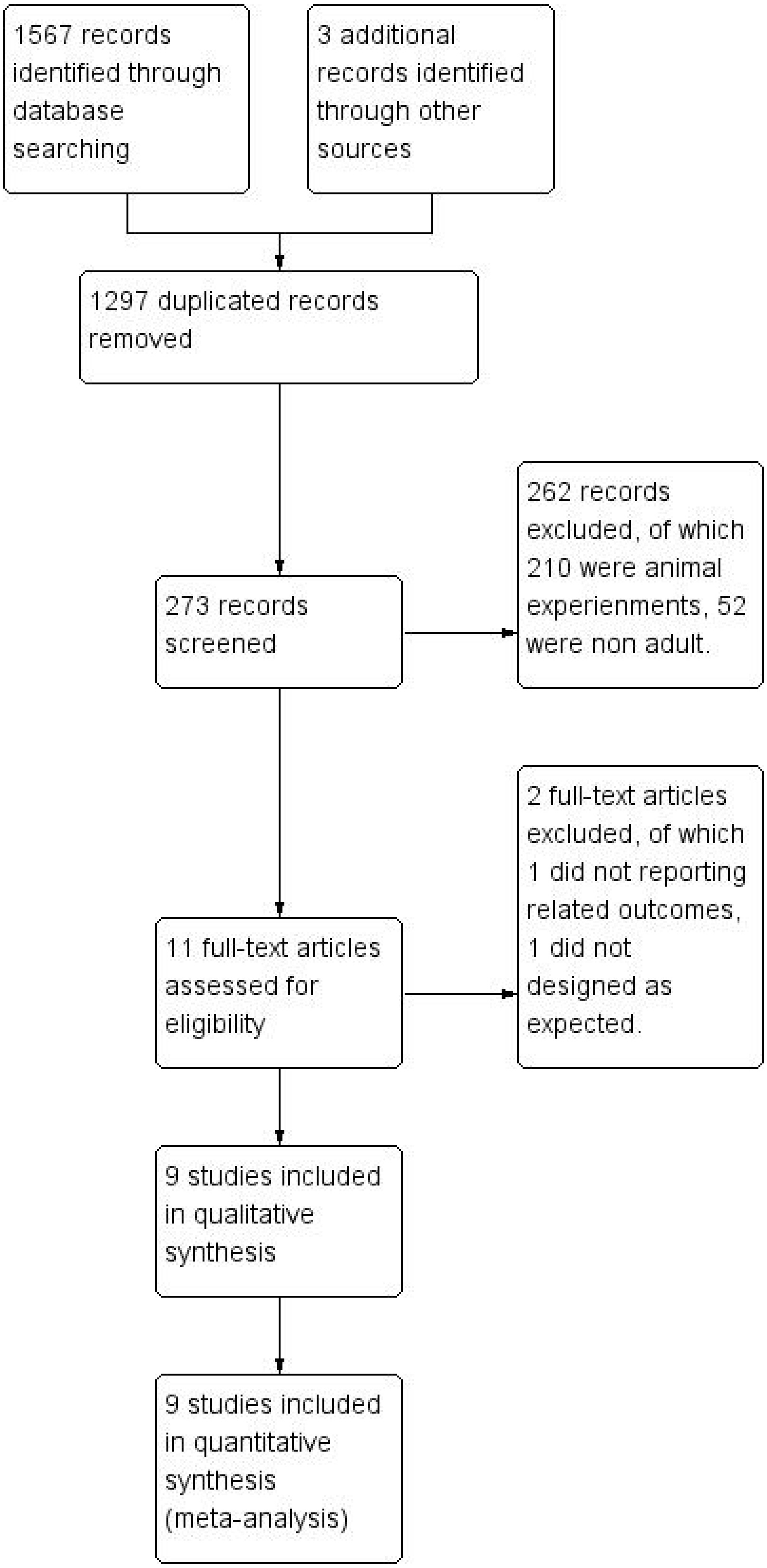

Figure 2s Risk of bias graph

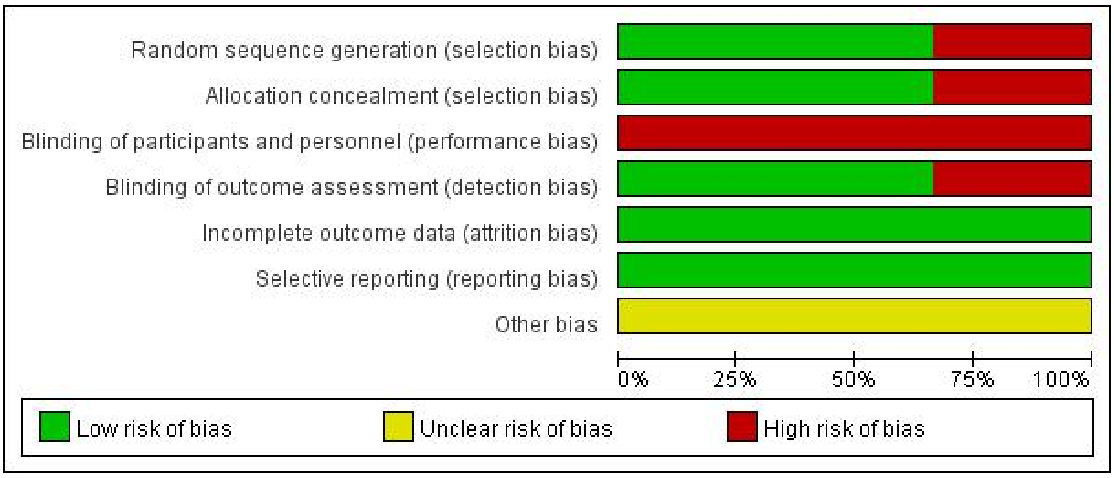

Figure3s Risk of bias summary

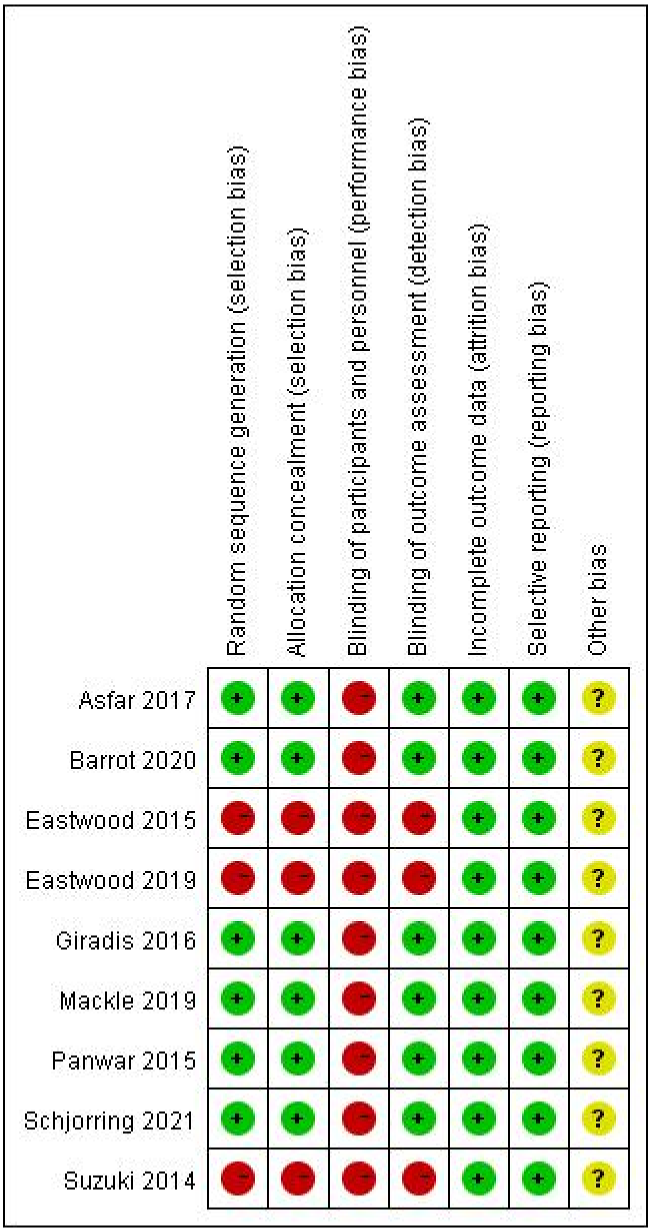

Figure 4s ICU mortality

Study or Subgroup	conservative Events	conservative Total	conventional Events	conventional Total	Weight	Risk Ratio M-H, Random, 95% CI
Barrot 2020	36	99	27	102	28.3%	1.37 [0.91, 2.08]
Eastwood 2016	15	50	16	50	22.2%	0.94 [0.52, 1.68]
Eastwood 2019	4	298	1	245	3.4%	3.29 [0.37, 29.23]
Giradis 2016	25	216	44	218	26.9%	0.57 [0.36, 0.90]
Panwar 2015	13	52	12	51	19.2%	1.06 [0.54, 2.10]
**Total (95% CI)**		**715**		**666**	**100.0%**	**0.98 [0.64, 1.49]**
Total events	93		100			

Heterogeneity: Tau² = 0.12; Chi² = 9.17, df = 4 (P = 0.06); I² = 56%
Test for overall effect: Z = 0.10 (P = 0.92)

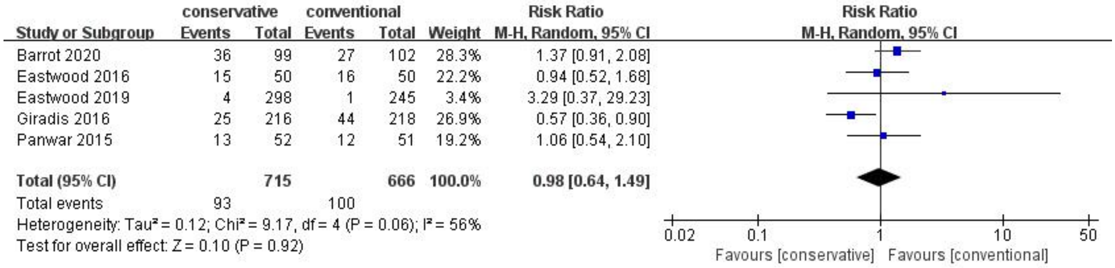

Figure 5s Hospital mortality

Study or Subgroup	conservative Events	conservative Total	conventional Events	conventional Total	Weight	Risk Ratio M-H, Random, 95% CI
Eastwood 2016	28	50	27	50	45.9%	1.04 [0.73, 1.48]
Eastwood 2019	5	298	1	245	3.9%	4.11 [0.48, 34.95]
Giradis 2016	52	216	74	218	50.3%	0.71 [0.53, 0.96]
**Total (95% CI)**		**564**		**513**	**100.0%**	**0.90 [0.59, 1.39]**
Total events	85		102			

Heterogeneity: Tau² = 0.07; Chi² = 4.70, df = 2 (P = 0.10); I² = 57%
Test for overall effect: Z = 0.46 (P = 0.65)

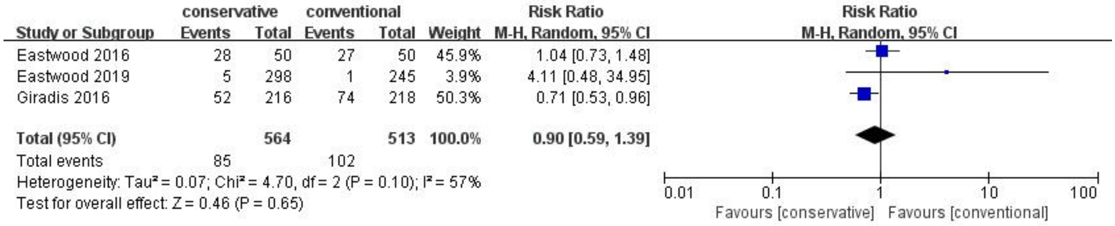

Figure 6s 28 day mortality

Study or Subgroup	conservative Events	conservative Total	conventional Events	conventional Total	Weight	Risk Ratio M-H, Fixed, 95% CI
Asfar 2017	77	217	93	217	57.4%	0.83 [0.65, 1.05]
Barrot 2020	34	99	27	102	16.4%	1.30 [0.85, 1.98]
Eastwood 2016	27	50	26	50	16.0%	1.04 [0.72, 1.50]
Suzuki 2014	9	54	16	51	10.2%	0.53 [0.26, 1.09]
**Total (95% CI)**		**420**		**420**	**100.0%**	**0.91 [0.76, 1.08]**
Total events	147		162			

Heterogeneity: Chi² = 5.95, df = 3 (P = 0.11); I² = 50%
Test for overall effect: Z = 1.07 (P = 0.28)

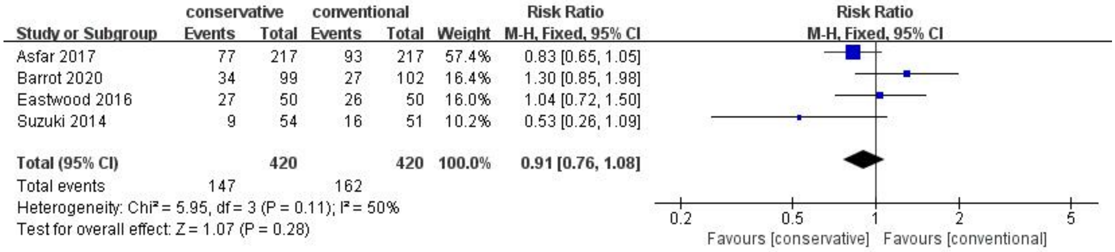

Figure 7s 90 day mortality

Study or Subgroup	conservative Events	conservative Total	conventional Events	conventional Total	Weight	Risk Ratio M-H, Random, 95% CI
Asfar 2017	90	217	104	217	19.5%	0.87 [0.70, 1.07]
Barrot 2020	44	99	31	102	8.3%	1.46 [1.01, 2.11]
Mackle 2019	166	479	156	480	23.9%	1.07 [0.89, 1.27]
Panwar 2015	21	52	19	51	5.1%	1.08 [0.67, 1.76]
Schjorring 2021	618	1441	613	1447	43.3%	1.01 [0.93, 1.10]
**Total (95% CI)**		**2288**		**2297**	**100.0%**	**1.03 [0.92, 1.15]**
Total events	939		923			

Heterogeneity: Tau² = 0.01; Chi² = 6.38, df = 4 (P = 0.17); I² = 37%
Test for overall effect: Z = 0.48 (P = 0.63)

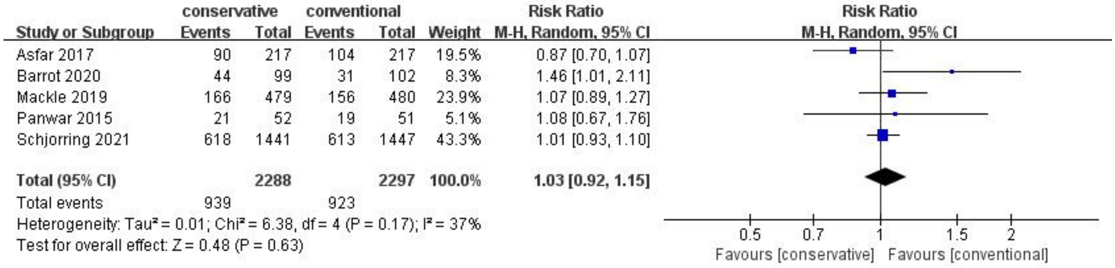

Figure 8s subgroup analysis about mortality in randomized controlled studies

Study or Subgroup	conservative Events	conservative Total	conventional Events	conventional Total	Weight	Risk Ratio M-H, Random, 95% CI
Asfar 2017	90	217	104	217	18.8%	0.87 [0.70, 1.07]
Barrot 2020	44	99	31	102	9.7%	1.46 [1.01, 2.11]
Giradis 2016	52	216	74	218	12.7%	0.71 [0.53, 0.96]
Mackle 2019	170	476	164	475	22.1%	1.03 [0.87, 1.23]
Panwar 2015	21	52	19	51	6.3%	1.08 [0.67, 1.76]
Schjorring 2021	618	1441	613	1447	30.6%	1.01 [0.93, 1.10]
**Total (95% CI)**		**2501**		**2510**	**100.0%**	**0.98 [0.86, 1.12]**
Total events	995		1005			

Heterogeneity: Tau² = 0.01; Chi² = 11.27, df = 5 (P = 0.05); I² = 56%
Test for overall effect: Z = 0.26 (P = 0.80)

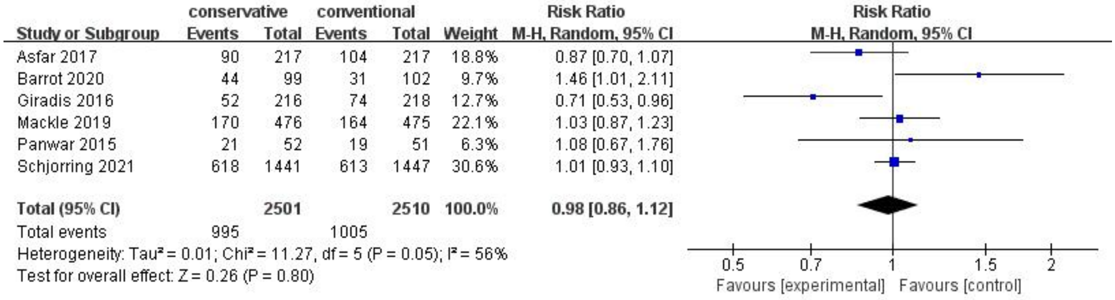

Figure 9s subgroup analysis according to the definition of conservative oxygen therapy

	conservative		conventional			Risk Ratio
Study or Subgroup	Events	Total	Events	Total	Weight	M-H, Fixed, 95% CI
Barrot 2020	44	99	31	102	4.3%	1.46 [1.01, 2.11]
Eastwood 2016	28	50	27	50	3.8%	1.04 [0.73, 1.48]
Eastwood 2019	6	298	3	245	0.5%	1.64 [0.42, 6.51]
Panwar 2015	21	52	19	51	2.7%	1.08 [0.67, 1.76]
Schjorring 2021	618	1441	613	1447	86.4%	1.01 [0.93, 1.10]
Suzuki 2014	9	54	16	51	2.3%	0.53 [0.26, 1.09]
**Total (95% CI)**		**1994**		**1946**	**100.0%**	**1.03 [0.95, 1.11]**
Total events	726		709			

Heterogeneity: Chi² = 7.38, df = 5 (P = 0.19); I² = 32%
Test for overall effect: Z = 0.65 (P = 0.52)

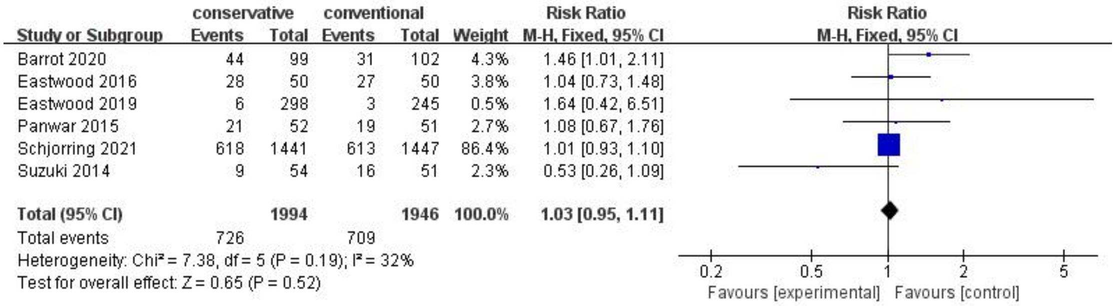

Supplement: Supplementary file 1 [file Data_Sheet_1.PDF]
